# The pandemic is gone but its consequences are here to stay: avascular necrosis following corticosteroids administration for severe COVID-19

**DOI:** 10.1186/s13018-024-04556-8

**Published:** 2024-02-12

**Authors:** Filippo Migliorini, Nicola Maffulli, Tapish Shukla, Riccardo D’Ambrosi, Mohit Singla, Abhishek Vaish, Raju Vaishya

**Affiliations:** 1 Department of Orthopaedic, Trauma, and Reconstructive Surgery, RWTH University Hospital, Pauwelsstraße 30, 52074 Aachen, Germany; 2 Department of Orthopedics and Trauma Surgery, Academic Hospital of Bolzano (SABES-ASDAA), Teaching Hospital of the Paracelsus Medical University, 39100 Bolzano, Italy; 3 Department of Medicine and Psychology, University of Rome “La Sapienza”, Rome, Italy; 4 Faculty of Medicine, School of Pharmacy and Bioengineering, Keele University, Stoke on Trent, ST4 7QB England; 5 Barts and the London School of Medicine and Dentistry, Centre for Sports and Exercise Medicine, Mile End Hospital, Queen Mary University of London, London, E1 4DG England; 6 Department of Orthopaedics and Joint Replacement Surgery, Indraprastha Apollo Hospitals Institutes of Orthopaedics, New Delhi, 110076 India; 7 Department of Orthopaedics, IRCCS Istituto Ortopedico Galeazzi, 20161 Milan, Italy; 8 Department of Orthopedics, PGIMS, Rohtak, Haryana 124001 India

**Keywords:** COVID-19, Steroids, Avascular necrosis, Osteonecrosis, Hip, Knee

## Abstract

**Background:**

In patients with COVID-19 infection and respiratory insufficiency, corticosteroid (CCS) administration is recommended. Among the wide range of complications and interactions, time-limited high-dose CCS administration might promote avascular necrosis (AVN) in a cumulative dose. This systematic review updated the current evidence and characterises the trend of AVN following time-limited high-dose CCS administration in patients who had severe COVID-19, discussing management strategies and outcomes.

**Methods:**

This systematic review was conducted according to the 2020 PRISMA statement. In October 2023, the following databases were accessed: PubMed, Web of Science, Google Scholar, and Scopus restricting the search to the years 2019 to 2023. All the clinical studies which investigated the association between time-limited high-dose CCS administration in patients with severe COVID-19 infection and AVN were accessed.

**Results:**

A total of 245 patients (9 studies) who experienced AVN following COVID-19 were included in the present investigation. 26% (63 of 245 included patients) were women. The mean age of the patients was 42.9 ± 17.7 years. Four studies focused on AVN of the hip and two on the knee, and the other studies included patients with AVN from mixed areas of the body (spine, pelvis, and shoulder). The mean time elapsed from COVID-19 infection to the development of symptomatic AVN was 79.4 ± 59.2 days (range, 14 to 166 days).

**Conclusion:**

It is possible that even time-limited high-dose CCS administration in patients with severe COVID-19 infection increased the incidence of AVN. The mean time elapsed from COVID-19 infection to the development of symptomatic AVN was approximately 80 days. Given the high risk of bias in all the included studies, the quality of recommendations of the present investigation is low, and no reliable conclusion can be inferred.

## Introduction

The Coronavirus disease-2019 (COVID-19) emerged in November 2019 in Wuhan, China, and was declared by the World Health Organisation (WHO) a pandemic a few months later [1, 2]. Other coronaviruses caused epidemics in the past decades, including Middle East Respiratory Syndrome (MERS) and Severe Acute Respiratory Syndrome (SARS) [3, 4]. The COVID-19 pandemic imposed healthcare systems worldwide with new challenges and considerable organisational efforts [5–10]. Often far-reaching process changes, such as the management of patient flows, had to be implemented in everyday clinical practice within a very short period [11–15]. The management of COVID-19 is complex and, to date, no shared guidelines exist [16–18]. In patients with COVID-19 infection and respiratory insufficiency, corticosteroids (CCS) administration is recommended [19–21]. CCS administration is indicated in patients receiving oxygen therapy or invasive mechanical ventilation [22–25]. CCS immunomodulates the acute cytokine storm, which is pivotal in the management of COVID-19 [22, 24]. However, depending on the timing of administration, the immunomodulation promoted by CCS might be beneficial (if hyperactivation had already occurred) or detrimental in the case of early immune response inhibition [26–28]. No benefit of CCS administration has been demonstrated in patients with no critical symptoms [22, 29, 30]. However, CCS administration is associated with several complications, including immunodeficiency, diabetes, hypertension, obesity, and thromboembolism [31–33]. Given the high rate of CCS administration and the onset of related complications, the European Society of Endocrinology (ESE) has recently commissioned an urgent clinical guidance document on CCS administration in a COVID-19 period [34]. Among this wide range of complications and interactions, time-limited high-dose CCS administration might promote avascular necrosis (AVN) in a cumulative dose [35–37]. AVN is characterised by the refractory and progressive compromise of bone architecture and vascularisation, which leads to morphological remodelling, premature osteoarthritis (OA), persistent pain, and loss of function [38–42]. This systematic review updated current evidence and characterised the trend of AVN following time-limited high-dose CCS administration in patients who had severe COVID-19, investigating the time elapsed from COVID-19 infection and the development of symptomatic AVN, and discussing management strategies and related outcomes.

## Methods

### Eligibility criteria

All the clinical studies which investigated the association between time-limited high-dose CCS administration in patients with severe COVID-19 infection and AVN were accessed. According to the author's language capabilities, articles in English, German, Italian, French and Spanish were eligible. Studies with levels I to IV of evidence, according to the Oxford Centre of Evidence-Based Medicine [43], were considered. Posters, abstracts, comments, editorials, opinions, and reviews were not eligible. Only investigations which reported quantitative data on the association between time-limited high-dose CCS administration in patients with severe COVID-19 infection and AVN were eligible.

### Search strategy

This systematic review was conducted according to the Preferred Reporting Items for Systematic Reviews and Meta-Analyses: the 2020 PRISMA statement [44]. The PICO algorithm was preliminarily set out:

- P (Problem): COVID-19 infection
- I (Intervention): time-limited high-dose CCS administration
- C (Comparison): AVN
- O (Outcomes): risk factors, incidence, management, outcome.

In October 2023, the following databases were accessed: PubMed, Web of Science, Google Scholar, and Scopus. The search was restricted to the years 2019 to 2023. The following keywords were used in combination using the Boolean operators AND/OR: *(COVID* OR *COVID-19* OR *pandemic)* AND *(avascular osteonecrosis* OR *aseptic osteonecrosis* OR *osteonecrosis)* AND *(pain* OR *outcome* OR *incidence* OR *prevalence* OR *symptoms)* AND* (steroids* OR *corticosteroids* OR *cortisone* OR *dexamethasone* OR *hydrocortisone* OR *prednisolone* OR *betamethasone)*.

### Selection and data collection

Two authors (FM & TS) independently performed the database search. All the resulting titles were screened, and if suitable, the abstract was accessed. The full text of the abstracts which matched the topic was accessed. The bibliography of the full-text articles was also screened for inclusion. Any disagreements were discussed and settled by a third senior author (NM).

### Data items

Two authors (FM & TS) independently performed data extraction. Studies generalities (author, year, name of the journal, nature of the study design, and purpose of the study) and data on patient demographics (study size, number of women, and mean age of the patients) were retrieved. Data on the type of CCS, severity of infection, main conclusion, and the time elapsed from COVID-19 infection and the diagnosis of symptomatic AVN were collected.

### Study risk of bias assessment

Two reviewers (TS & RDA) independently performed the methodological quality assessment of the extracted studies. Disagreements were solved by a third senior author (NM). The study risk of bias assessment was conducted in accordance with the guidelines in the Cochrane Handbook for Systematic Reviews of Interventions [45]. The risk of bias of the software Review Manager 5.3 (The Nordic Cochrane Collaboration, Copenhagen) was used for Randomised Control Trials (RCTs). The Risk of Bias in Nonrandomised Studies of Interventions (ROBINS-I) tool was used for non-RCTs [46].

### Statistical analysis

For descriptive statistics, the SPSS software version 25 (IBM, International Business Machines Corp, Armonk, US) was used.

## Results

### Study selection

**Fig. 1:**
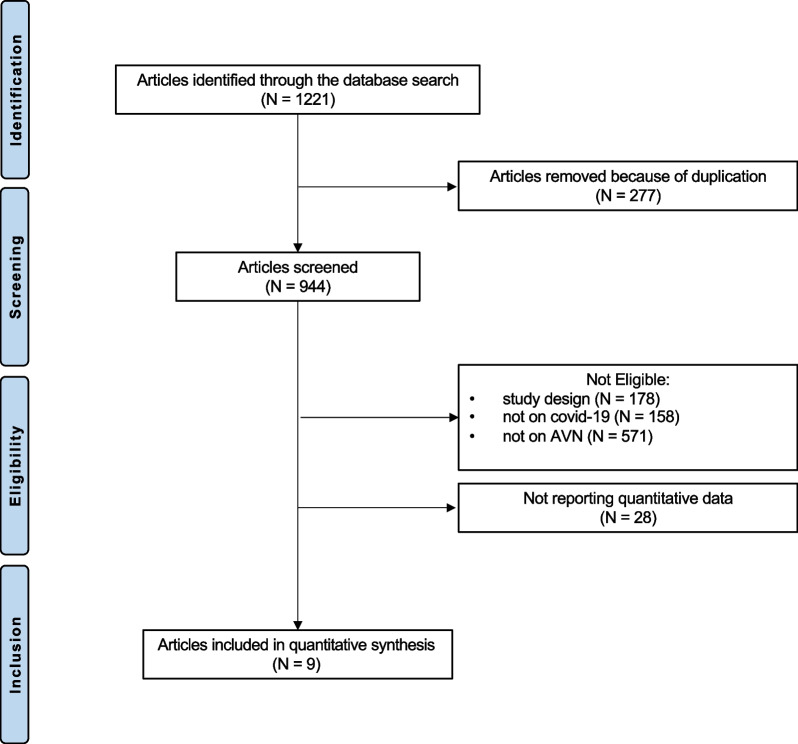
Flow chart of the literature search

A total of 1221 articles resulted from the literature search. Of them, 277 were duplicates. A further 757 articles were excluded for the reasons: no reported data on COVID-19 patients (*N* = 158), no reported data on AVN (*N* = 571), type of study (*N* = 178), did not report quantitative data on the outcome of interest (*N* = 28). Finally, 9 articles were included in the present study. The flow chart of the literature search is shown in Fig. 1.

### Study risk of bias assessment

The ROBINS-I score evidenced an overall high risk of bias. Most studies were retrospective case series and no RCT was available, with a high risk of selection, detection and performance biases. All the risks of bias investigated in the ROBINS-I score were serious and critical (Table 1).

**Table 1:** ROBINS-I of the included studies

Study	Bias due to confounding	Bias in selection of participants into the study	Bias in classification of interventions	Bias due to deviations from intended intervention	Bias due to missing data	Bias in measurement of outcomes	Bias in selection of the reported results	Overall risk of bias judgement
Agarwala et al., [47]	Critical	Critical	Critical	Critical	Critical	Critical	Critical	Critical
Agarwala et al., [48]	Serious	Serious	Serious	Serious	Serious	Serious	Serious	Serious
Alkindi et al., [49]	Critical	Serious	Serious	Critical	Critical	Critical	Critical	Critical
Chacko et al., [50]	Serious	Critical	Critical	Critical	Critical	Critical	Critical	Critical
Daltro et al., [51]	Serious	Serious	Serious	Serious	Serious	Serious	Serious	Serious
Ghosh et al., [52]	Critical	Serious	Critical	Critical	Critical	Serious	Critical	Critical
Kachewar et al., [53]	Critical	Critical	Serious	Critical	Critical	Critical	Serious	Critical
Panin et al., [54]	Serious	Serious	Serious	Serious	Serious	Serious	Serious	Serious
Sulewski et al., [55]	Serious	Serious	Serious	Serious	Serious	Serious	Serious	Serious

### Study characteristics and results of individual studies

A total of 245 patients who experienced AVN following COVID-19 were included in the present investigation. 26% (63 of 245 included patients) were women. The mean age of the patients was 42.9 ± 17.7 years. Four studies [50, 51, 53, 54], overall involving 228 patients, focused on AVN of the hip, and two studies (5 patients) [47, 48] focused exclusively on the knee. The other studies included patients with AVN deriving from mixed areas of the body (spine, pelvis, shoulder) [49, 52, 55]. Study generalities and data of the patients are shown in Table 2.

**Table 2:** Generalities of the included studies (AVN: avascular necrosis; CCS: corticosteroids)

Author and year	Journal	Study design	Patients (*n*)	Joint	Female (%)	Age (mean)
Agarwala et al., [47]	BMJ Case Reports	Case reports	3	Knee	0	37
Agarwala et al., [48]	BMJ Case Reports	Case series	2	Knee	50	
Alkindi et al., [49]	European Medical Journal	Case report	1	Hip/knee	0	29
Chacko et al., [50]	International Journal of Research in Orthopaedics	Case report	1	Hip	0	23
Daltro et al., [51]	Journal of Regenerative Biology and Medicine	Case report	23	Hip	34	44
Ghosh et al., [52]	Indian Journal of Radiology and Imaging	Case reports	1	Spine/hip/pelvis	0	72
Kachewar et al., [53]	Indian Journal of Musculoskeletal Radiology	Retrospective	200	Hip	23	
Panin et al., [54]	Traumatology And Orthopaedics of Russia	Case series	4	Hip	50	34
Sulewski et al., [55]	Medicina	Case series	10	Spine/hip/pelvis/shoulder/knee	60	61

Table 3 reports the aims of the studies and the main results of the included studies. The mean time elapsed from COVID-19 infection to the development of symptomatic AVN was 79.4 ± 59.2 days (range, 14 to 166 days).

**Table 3:** Main findings of the included studies (AVN: avascular necrosis; CCS: corticosteroids; MRI: magnetic resonance imaging)

Author and year	Time to AVN (*days*)	Aim of the study	Main results of the study
Agarwala et al., [47]	49	To report early development of AVN in post-COVID patients	COVID-19-related treatment may induce AVN in approximately two months following the infection
Agarwala et al., [48]	73	To investigate the possible knee AVN as part of long COVID-19 syndrome	After COVID-19, patients are more susceptible to osteonecrosis. Bisphosphonates are effective in early stages of osteonecrosis of knee
Alkindi et al., [49]	166	To report the case of bilateral AVN of femoral heads and proximal tibias after 8 weeks after suffering from severe COVID-19	It is possible that AVN and reactive arthritis are directly linked to the COVID-19 and some superimposed exacerbated it
Chacko et al., [50]	56	To report a case of bilateral femoral head AVN in a post-COVID-19 male patient	There is an increased risk of osteonecrosis of femoral head in COVID-19 case due to hypercoagulability state associated with COVID-19
Daltro et al., [51]	133	To report a case of patient infected with COVID-19 who developed femoral head AVN	CCS and association of hypercoagulability mechanisms related to COVID-19 and AVN
Ghosh et al., [52]	30	To report a case of a man with COVID-19-associated vertebral, femoral and pelvic AVN	The findings are highly suggestive of COVID-19-associated AVN
Kachewar et al., [53]		To study the MRI images of COVID-19 infected patients for spectrum of femoral head AVN	6% incidence of AVN was seen in patients who developed hip pain after being treated for COVID-19
Panin et al., [54]	114	To report a series of patients treated COVID-19 with bilateral femoral heads AVN	Factors associated with COVID-19 and related treatment can influence the development of AVN
Sulewski et al., [55]	14	To report ten patients with AVN following COVID-19	COVID-19 can have a negative impact on bones at 1 to 3 weeks

## Discussion

According to the main findings of the present systematic review, there is evidence of increased incidence of AVN after the use of time-limited high-dose CCS therapy in patients with COVID-19 infection. The mean time elapsed from COVID-19 infection to the development of symptomatic AVN was approximately 80 days. Given the high risk of bias in all the included studies, the quality of recommendations of the present investigation is low, and no reliable conclusion can be inferred. However, after the 2003 SARS outbreak, up to 23% of patients receiving CCS developed AVN of the femoral head [56–58]; if the incidence of AVN peaks in these COVID-19 cases at those levels, a pandemic of AVN might happen, considering the massive outbreak of COVID-19 infection across the globe.

Several clinical investigations have explored the time-limited high-dose CCS administration in patients with COVID-19 infection receiving oxygen therapy or invasive mechanical ventilation (Table 4). However, no study reported cases of AVN among time-limited high-dose CCS-treated patients. On the other hand, we were able to identify for inclusion only articles with low levels of evidence in the present study. In this context, no high-quality recommendations can be inferred. Further, high-quality investigations and multi-centre studies are required to establish the actual incidence of AVN following time-limited high-dose CCS therapy in patients with COVID-19 infection.

**Table 4:** Clinical investigations exploring the time-limited high-dose CCS administration in patients with COVID-19 infection receiving oxygen therapy or invasive mechanical ventilation

Author, year	Country	Journal	Study Design	Sample Size	Treatment
Galvez-Romero et al. [59]	Mexico	*J Intern Med*	Open-label, non-RCT	209	Methylprednisolone or Prednisone
Fernández-Cruz et al. [60]	Spain	*Antimicrob Agents Chemother*	Retrospective, controlled	463	Methylprednisolone
Horby et al. [61]	United Kingdom	*N Engl J Med*	Prospective, controlled, open-label	6425	Dexamethasone
Keller et al. [62]	USA	*J Hosp Med*	Retrospective	1806	Glucocorticoids (not specified)
Mikulska et al. [63]	Italy	*PLoS One*	Retrospective	196	Methylprednisolone
Murohashi et al. [64]	Japan	*Respir Investig*	Retrospective	11	Methylprednisolone
Obata et al. [65]	America	*Jpn J Infect Dis Action Epub*	Retrospective	226	Dexamethasone, Prednisolone
Rana et al. [66]	Pakistan	*Cureus*	Retrospective	60	Dexamethasone or Methylprednisolone
Rodríguez-Molinero et al. [67]	Spain	*Med Clin (Barc)*	Retrospective	418	Methylprednisolone
Spagnuolo et al. [68]	Italy	*Sci Rep*	Retrospective	280	Methylprednisolone
Xiaofan Lu et al. [69]	China	*N Engl J Med*	Retrospective	244	Hydrocortisone
Yan et al. [70]	China	*Biomed Pharmacother*	Retrospective	308	Methylprednisolone
Zhu et al. [71]	China	*World J Clin Cases*	Retrospective	102	Methylprednisolone

The analysis included 13 articles for a total of 10,748 patients treated with steroids for COVID-19. Eleven studies were retrospective, one open-label non-RCT, and one prospective controlled, open-label trial. The steroids used varied from Dexamethasone, Prednisone, or Methylprednisolone. No case of AVN among time-limited high-dose CCS-treated patients was reported

Previous evidence demonstrated that the use of CCS and their association with AVN is related to cumulative dose [35, 72–74]. However, there is no consensus on their safety threshold. In a recent RCT, the administration of Dexamethasone 6 mg daily for 10 days (600 mg of cumulative dose) in COVID-19 patients was associated with a reduction of the need for mechanical ventilation and 28-day mortality [25, 61]. This dose regime is well below the cumulative dose thresholds described in much of the literature (cumulative dose of 2 g prednisone or its equivalent) [75, 76]. However, there is growing evidence that, if the clinical conditions deteriorate or patients are admitted to critical care, greater pulse doses of CCS (up to 125 mg per day) should be administered [77–80]. However, AVN has been reported even at much lower dose thresholds [81–83]. In a recent meta-analysis of 10 studies (1137 recovered patients with SARS) the risk of AVN was low if the cumulative dose of CCS (methylprednisolone) was ≤ 5 g. Greater cumulative doses of 5 g to 10 g were at high risk for AVN [84].

Osteonecrosis of the femoral head is the most common type of AVN. The management of this condition is challenging and controversial, with unpredictable results [85–87]. A recent systematic review of 88 articles (6112 procedures) evidenced that men, longer symptom duration, and greater pain were negative prognostic factors for AVN of the femoral head [88]. In the early phases of AVN of the femoral head, core decompression, percutaneous drilling and micro-drilling are performed to increase blood supply into the necrotic bone area [89]. However, their efficacy is limited [90, 91]. Core decompression augmented with bone marrow-derived mesenchymal stem cells has been proposed to improve the bone healing activity [92–106]. In a recent meta-analysis of 7 RCTs (579 patients), core decompression combined with bone marrow mesenchymal stem cells showed a lower rate of total hip arthroplasty compared to core decompression in isolation [107]. Additional hip preserving strategies include non-vascularized/vascularized autograft/allograft/synthetic bone grafting, high femoral osteotomies, and drug administration (i.e. bisphosphonates) [108–115]. A recent Bayesian network meta-analysis of 32 clinical trials (2367 procedures) compared several conservative and operative strategies [116]. Conservative management and isolated core decompression were associated with the lowest success rate [116]. Core decompression combined with autologous bone grafting and enhanced with bone marrow concentrate was effective in reducing the rate of failure and progression to hip arthroplasty [116]. Despite these surgical options, AVN of the femoral head often progresses to subchondral fractures, femoral head collapse, and painful OA [117–120]. In those patients, total hip arthroplasty is considered the last resort in the management of AVN [121].

We acknowledge several limitations of this study. Given the high risk of bias in all the included studies, the quality of recommendations of the present investigation is low, and no definite conclusions can be inferred. Given the lack of information on administration protocol and timing of CCS, additional subgroup analyses were not performed. In one included study, only 6% of the entire cohort of patients who had severe COVID-19 developed AVN following time-limited high-dose CCS administration [53]. The definition of severe COVID was not clearly stated in most articles, which poses limits to properly defining the study population. The current literature will benefit future high-quality investigations. Most studies were case reports, with a limited number of patients and not using validated patient-reported outcome measures (PROMs). The surgical procedures and rehabilitation protocols were also biased in most studies, as were general health measures. COVID-19-associated therapies and severity, comorbidities and patient characteristics were also seldom described in most studies. Most patients affected by AVN are young and active, which makes the management of such a potentially devastating condition troublesome. The current treatment strategies are limited, with unpredictable results; despite some new treatment modalities having been introduced with promising results [122, 123], prevention, early diagnosis, and early treatment remain the way to manage AVN. The type of CCS used to manage respiratory insufficiency and the severity of the COVID-19 infection were not reported by most authors. Moreover, the severity and progression of AVN were not classified using validated classification, such as the Association Research Circulation Osseous (ARCO) for the femoral head.

## Conclusion

Some evidence supports that even time-limited high-dose CCS administration in patients with COVID-19 infection increased the incidence of AVN. The mean time elapsed from COVID-19 infection to the development of symptomatic AVN was approximately of 80 days. Given the high risk of bias in all the included studies, the quality of recommendations of the present investigation is low, and no reliable conclusion can be inferred.

## Data Availability

The datasets generated during and/or analysed during the current study are available throughout the manuscript.

## Abbreviations

AVN: Avascular necrosis
OA: Osteoarthritis
CCS: Corticosteroids
PROMs: Patients-reported outcome measures
ARCO: Association Research Circulation Osseous

## References

[1] Nishiura H, Jung SM, Linton NM, Kinoshita R, Yang Y, Hayashi K, Kobayashi T, Yuan B, Akhmetzhanov AR (2020). The extent of transmission of novel coronavirus in Wuhan, China, 2020. J Clin Med.

[2] Yang Y, Peng F, Wang R, Yange M, Guan K, Jiang T, Xu G, Sun J, Chang C (2020). The deadly coronaviruses: The 2003 SARS pandemic and the 2020 novel coronavirus epidemic in China. J Autoimmun.

[3] Leung TYM, Chan AYL, Chan EW, Chan VKY, Chui CSL, Cowling BJ, Gao L, Ge MQ, Hung IFN, Ip MSM, Ip P, Lau KK, Lau CS, Lau LKW, Leung WK, Li X, Luo H, Man KKC, Ng VWS, Siu CW, Wan EYF, Wing YK, Wong CSM, Wong KHT, Wong ICK (2020). Short- and potential long-term adverse health outcomes of COVID-19: a rapid review. Emerg Microbes Infect.

[4] Testa G, Sapienza M, Rabuazzo F, Culmone A, Valenti F, Vescio A, Pavone V (2021). Comparative study between admission, orthopaedic surgery, and economic trends during COVID-19 and non-COVID-19 pandemic in an Italian tertiary hospital: a retrospective review. J Orthop Surg Res.

[5] Migliorini F, Weber CD, Pappalardo G, Schenker H, Hofmann UK, Eschweiler J, Hildebrand F (2022). Orthopaedic, trauma surgery, and COVID-2019 pandemic: clinical panorama and future prospective in Europe. Eur J Trauma Emerg Surg.

[6] Atzrodt CL, Maknojia I, McCarthy RDP, Oldfield TM, Po J, Ta KTL, Stepp HE, Clements TP (2020). A Guide to COVID-19: a global pandemic caused by the novel coronavirus SARS-CoV-2. FEBS J.

[7] Harter M, Bremer D, Scherer M, von dem Knesebeck O, Koch-Gromus U (2020). Impact of COVID-19-pandemic on clinical care, work flows and Staff at a University Hospital: results of an Interview-study at the UKE. Gesundheitswesen.

[8] Wong J, Goh QY, Tan Z, Lie SA, Tay YC, Ng SY, Soh CR (2020). Preparing for a COVID-19 pandemic: a review of operating room outbreak response measures in a large tertiary hospital in Singapore. Can J Anaesth.

[9] Migliorini F, Torsiello E, Spiezia F, Oliva F, Tingart M, Maffulli N (2021). Association between HLA genotypes and COVID-19 susceptibility, severity and progression: a comprehensive review of the literature. Eur J Med Res.

[10] de Girolamo L, Peretti GM, Maffulli N, Brini AT (2020). COVID-19-the real role of NSAIDs in Italy. J Orthop Surg Res.

[11] CfMMSCfMMsCrR-oftpn-en-C-hpIA. at: https://www.cms.gov/files/document/COVID-flexibility-reopen-essential-non-COVID-services.pdf. Accessed in March 2021

[12] Giordano L, Cipollaro L, Migliorini F, Maffulli N (2021). Impact of COVID-19 on undergraduate and residency training. Surgeon.

[13] Luceri F, Morelli I, Accetta R (2020). Italy and COVID-19: the changing patient flow in an orthopedic trauma center emergency department. J Orthop Surg Res.

[14] Wignall A, Giannoudis V, De C (2021). The impact of COVID-19 on the management and outcomes of patients with proximal femoral fractures: a multi-centre study of 580 patients. J Orthop Surg Res.

[15] Bagherifard A, Arasteh P, Salehpour M, Zadeh HS, Mazhar FN, Ghandhari H, Bahaeddini MR, Tabrizian P, Askari A (2021). COVID-19 among patients with orthopedic surgery: our experience from the Middle East. J Orthop Surg Res.

[16] Hu B, Guo H, Zhou P, Shi ZL (2021). Characteristics of SARS-CoV-2 and COVID-19. Nat Rev Microbiol.

[17] Mehandru S, Merad M (2022). Pathological sequelae of long-haul COVID. Nat Immunol.

[18] Alturkistany A, Abduljabbar FH, Alhelal F, Dajim NB, Khalifah S, Konbaz F, Aleissa S, Al-Habib A, Kattan M, Alqahtani Y, Alatassi R (2020). The Saudi spine society guidelines on spinal surgery during the COVID-19 pandemic. J Orthop Surg Res.

[19] Parasher A (2021). COVID-19: current understanding of its pathophysiology, clinical presentation and treatment. Postgrad Med J.

[20] Raman R, Patel KJ, Ranjan K (2021). COVID-19: unmasking emerging SARS-CoV-2 variants, vaccines and therapeutic strategies. Biomolecules.

[21] Chifu I, Detomas M, Dischinger U, Kimpel O, Megerle F, Hahner S, Fassnacht M, Altieri B (2021). Management of patients with glucocorticoid-related diseases and COVID-19. Front Endocrinol.

[22] RECOVERY Coll Group, Horby P, Lim WS, Emberson JR, Mafham M, Bell JL, al. e (2021) Dexamethasone in hospitalized patients with COVID-19. N Engl J Med. 384(8):693–704. 10.1056/NEJMoa2021436 10.1056/NEJMoa2021436 PMC7383595 32678530

[23] Berlin DA, Gulick RM, Martinez FJ (2020). Severe COVID-19. N Engl J Med.

[24] Chifu I, Detomas M, Dischinger U, Kimpel O, Megerle F, Hahner S, Fassnacht M, Altieri B (2021). Management of patients with glucocorticoid-related diseases and COVID-19. Front Endocrinol.

[25] van Paassen J, Vos JS, Hoekstra EM, Neumann KMI, Boot PC, Arbous SM (2020). Corticosteroid use in COVID-19 patients: a systematic review and meta-analysis on clinical outcomes. Crit Care.

[26] Chrousos GP (1995). The hypothalamic-pituitary-adrenal axis and immune-mediated inflammation. N Engl J Med.

[27] Halpin DMG, Criner GJ, Papi A, Singh D, Anzueto A, Martinez FJ, Agusti AA, Vogelmeier CF (2021). Global initiative for the diagnosis, management, and prevention of chronic obstructive lung disease. the 2020 GOLD science committee report on COVID-19 and chronic obstructive pulmonary disease. Am J Respir Crit Care Med.

[28] Almeida MQ, Mendonca BB (2020). Adrenal insufficiency and glucocorticoid use during the COVID-19 pandemic. Clinics.

[29] Denneny EK, Garthwaite HS, Heightman MJ, Porter JC (2021). A role for steroids in COVID-19-associated pneumonitis at six-week follow-up?. Ann Am Thorac Soc.

[30] Sahu AK, Mathew R, Bhat R, Malhotra C, Nayer J, Aggarwal P, Galwankar S (2021). Steroids use in non-oxygen requiring COVID-19 patients: a systematic review and meta-analysis. QJM.

[31] Guarnotta V, Ferrigno R, Martino M, Barbot M, Isidori AM, Scaroni C, Ferrante A, Arnaldi G, Pivonello R, Giordano C (2021). Glucocorticoid excess and COVID-19 disease. Rev Endocr Metab Disord.

[32] Pivonello R, Isidori AM, De Martino MC, Newell-Price J, Biller BM, Colao A (2016). Complications of Cushing's syndrome: state of the art. Lancet Diabetes Endocrinol.

[33] Arnaldi G, Angeli A, Atkinson AB, Bertagna X, Cavagnini F, Chrousos GP, Fava GA, Findling JW, Gaillard RC, Grossman AB, Kola B, Lacroix A, Mancini T, Mantero F, Newell-Price J, Nieman LK, Sonino N, Vance ML, Giustina A, Boscaro M (2003). Diagnosis and complications of Cushing's syndrome: a consensus statement. J Clin Endocrinol Metab.

[34] Newell-Price J, Nieman LK, Reincke M, Tabarin A (2020). Endocrinology in the time of covid-19: management of Cushing's syndrome. Eur J Endocrinol.

[35] Guo KJ, Zhao FC, Guo Y, Li FL, Zhu L, Zheng W (2014). The influence of age, gender and treatment with steroids on the incidence of osteonecrosis of the femoral head during the management of severe acute respiratory syndrome: a retrospective study. Bone Jt J.

[36] Weinstein RS (2012). Glucocorticoid-induced osteonecrosis. Endocrine.

[37] Mont MA, Pivec R, Banerjee S, Issa K, Elmallah RK, Jones LC (2015). High-dose corticosteroid use and risk of hip osteonecrosis: meta-analysis and systematic literature review. J Arthroplasty.

[38] Beverly MC, Murray DW (2021). Subchondral physiology and vasculo-mechanical factors in load transmission and osteoarthritis. Bone Jt Res.

[39] Addai D, Zarkos J, Pettit M, Sunil Kumar KH, Khanduja V (2021). Outcomes following surgical management of femoroacetabular impingement: a systematic review and meta-analysis of different surgical techniques. Bone Jt Res.

[40] Husain R, Nesbitt J, Tank D, Verastegui MO, Gould ES, Huang M (2020). Spontaneous osteonecrosis of the knee (SONK): the role of MR imaging in predicting clinical outcome. J Orthop.

[41] Rizkalla J, Jeffers M, Salama P, Rizkalla M (2018). Electromagnetic simulation for diagnosing damage to femoral neck vasculature: a feasibility study. J Orthop.

[42] Goto K, Aoyama T, Toguchida J, Kuroda Y, Kawai T, Okuzu Y, Matsuda S (2021). Ten-year results of mesenchymal stromal cell transplantation augmented with vascularised bone grafts for advanced osteonecrosis of the femoral head. J Orthop.

[43] Howick J CI, Glasziou P, Greenhalgh T, Carl Heneghan, Liberati A, Moschetti I, Phillips B, Thornton H, Goddard O, Hodgkinson M. The 2011 Oxford CEBM levels of evidence. Oxford Centre for Evidence-Based Medicine; 2011.

[44] Page MJ, McKenzie JE, Bossuyt PM, Boutron I, Hoffmann TC, Mulrow CD, Shamseer L, Tetzlaff JM, Akl EA, Brennan SE, Chou R, Glanville J, Grimshaw JM, Hrobjartsson A, Lalu MM, Li T, Loder EW, Mayo-Wilson E, McDonald S, McGuinness LA, Stewart LA, Thomas J, Tricco AC, Welch VA, Whiting P, Moher D (2021). The PRISMA 2020 statement: an updated guideline for reporting systematic reviews. BMJ.

[45] Cumpston M, Li T, Page MJ, Chandler J, Welch VA, Higgins JP, Thomas J. Updated guidance for trusted systematic reviews: a new edition of the Cochrane Handbook for Systematic Reviews of Interventions. Cochrane Database Syst Rev. 2019;10:ED000142. 10.1002/14651858.ED000142 10.1002/14651858.ED000142 PMC10284251 31643080

[46] Sterne JA, Hernan MA, Reeves BC, Savovic J, Berkman ND, Viswanathan M, Henry D, Altman DG, Ansari MT, Boutron I, Carpenter JR, Chan AW, Churchill R, Deeks JJ, Hrobjartsson A, Kirkham J, Juni P, Loke YK, Pigott TD, Ramsay CR, Regidor D, Rothstein HR, Sandhu L, Santaguida PL, Schunemann HJ, Shea B, Shrier I, Tugwell P, Turner L, Valentine JC, Waddington H, Waters E, Wells GA, Whiting PF, Higgins JP (2016). ROBINS-I: a tool for assessing risk of bias in non-randomised studies of interventions. BMJ.

[47] Agarwala SR, Vijayvargiya M, Pandey P (2021). Avascular necrosis as a part of 'long COVID-19'. BMJ Case Rep.

[48] Agarwala SR, Vijayvargiya M, Sawant T (2022). Secondary osteonecrosis of the knee as a part of long COVID-19 syndrome: a case series. BMJ Case Rep.

[49] Alkindi F, Nokhatha SA, Alseiari K, Alnaqb KA (2022). Reactive hip arthritis and avascular necrosis after severe COVID-19 infection: a case report and comprehensive review of literature. Eur Med J.

[50] Ankith C, Babu M, Dibin KT (2021). Osteonecrosis of bilateral femoral head in a post COVID-19 patient: case report. Int J Res Orthop.

[51] Daltro G, Franco BA, Veiga D, Faleiro T, Lima V, Vitório F (2021). Osteonecrosis development post COVID-19 infection. J Regen Biol Med.

[52] Ghosh S, Gupta SS, Mehta N, Khodaiji S (2021). COVID-19-associated bone marrow necrosis-a case report. Indian J Radiol Imaging.

[53] Kachewar SG, Kachewar SS (2022). MRI spectrum of avascular necrosis of femoral head in patients treated for COVID-19. Indian J Musculoskelet Radiol.

[54] Panin MA, Petrosyan AS, Hadjicharalambous KK, Boiko AV (2022). Avascular necrosis of the femoral head after COVID-19: a case series. Traumatol Orthop Russ.

[55] Sulewski A, Sieron D, Szyluk K, Dabrowski M, Kubaszewski L, Lukoszek D, Christe A (2021). Avascular necrosis bone complication after active COVID-19 infection: preliminary results. Medicina.

[56] Xie L, Liu Y, Fan B, Xiao Y, Tian Q, Chen L, Zhao H, Chen W (2005). Dynamic changes of serum SARS-coronavirus IgG, pulmonary function and radiography in patients recovering from SARS after hospital discharge. Respir Res.

[57] Griffith JF, Antonio GE, Kumta SM (2005). Osteonecrosis of hip and knee in patients with severe acute respiratory syndrome treated with steroids. Radiology.

[58] Chan MHM, Chan PKS, Griffith JF (2006). Steroid-induced osteonecrosis in severe acute respiratory syndrome: a retrospective analysis of biochemical markers of bone metabolism and corticosteroid therapy. Pathology.

[59] Galvez-Romero JL, Palmeros-Rojas O, Real-Ramirez FA, Sanchez-Romero S, Tome-Maxil R, Ramirez-Sandoval MP, Olivos-Rodriguez R, Flores-Encarnacion SE, Cabrera-Estrada AA, Avila-Morales J, Cortes-Sanchez V, Sarmiento-Padilla G, Tezmol-Ramirez SE, Aparicio-Hernandez D, Urbina-Sanchez MI, Gomez-Pluma MA, Cisneros-Mendez S, Rodriguez-Rivas DI, Reyes-Inurrigarro S, Cortes-Diaz G, Cruz-Delgado C, Navarro-Gonzalez J, Deveaux-Homs J, Pedraza-Sanchez S (2021). Cyclosporine A plus low-dose steroid treatment in COVID-19 improves clinical outcomes in patients with moderate to severe disease: A pilot study. J Intern Med.

[60] Fernandez-Cruz A, Ruiz-Antoran B, Munoz-Gomez A, Sancho-Lopez A, Mills-Sanchez P, Centeno-Soto GA, Blanco-Alonso S, Javaloyes-Garachana L, Galan-Gomez A, Valencia-Alijo A, Gomez-Irusta J, Payares-Herrera C, Morras-Torre I, Sanchez-Chica E, Delgado-Tellez-de-Cepeda L, Callejas-Diaz A, Ramos-Martinez A, Munez-Rubio E, Avendano-Sola C (2020). A retrospective controlled cohort study of the impact of glucocorticoid treatment in SARS-CoV-2 infection mortality. Antimicrob Agents Chemother.

[61] Group RC, Horby P, Lim WS, Emberson JR, Mafham M, Bell JL, Linsell L, Staplin N, Brightling C, Ustianowski A, Elmahi E, Prudon B, Green C, Felton T, Chadwick D, Rege K, Fegan C, Chappell LC, Faust SN, Jaki T, Jeffery K, Montgomery A, Rowan K, Juszczak E, Baillie JK, Haynes R, Landray MJ (2021). Dexamethasone in hospitalized patients with COVID-19. N Engl J Med.

[62] Keller MJ, Kitsis EA, Arora S, Chen JT, Agarwal S, Ross MJ, Tomer Y, Southern W (2020). Effect of systemic glucocorticoids on mortality or mechanical ventilation in patients with COVID-19. J Hosp Med.

[63] Mikulska M, Nicolini LA, Signori A, Di Biagio A, Sepulcri C, Russo C, Dettori S, Berruti M, Sormani MP, Giacobbe DR, Vena A, De Maria A, Dentone C, Taramasso L, Mirabella M, Magnasco L, Mora S, Delfino E, Toscanini F, Balletto E, Alessandrini AI, Baldi F, Briano F, Camera M, Dodi F, Ferrazin A, Labate L, Mazzarello G, Pincino R, Portunato F, Tutino S, Barisione E, Bruzzone B, Orsi A, Schenone E, Rosseti N, Sasso E, Da Rin G, Pelosi P, Beltramini S, Giacomini M, Icardi G, Gratarola A, Bassetti M (2020). Tocilizumab and steroid treatment in patients with COVID-19 pneumonia. PLoS ONE.

[64] Murohashi K, Hagiwara E, Kitayama T, Yamaya T, Higa K, Sato Y, Otoshi R, Shintani R, Okabayashi H, Ikeda S, Niwa T, Nakazawa A, Oda T, Okuda R, Sekine A, Kitamura H, Baba T, Komatsu S, Iwasawa T, Kaneko T, Ogura T (2020). Outcome of early-stage combination treatment with favipiravir and methylprednisolone for severe COVID-19 pneumonia: a report of 11 cases. Respir Investig.

[65] Obata R, Maeda T, Rizk D, Kuno T (2021). Increased secondary infection in COVID-19 patients treated with steroids in New York City. Jpn J Infect Dis.

[66] Rana MA, Hashmi M, Qayyum A, Pervaiz R, Saleem M, Munir MF, Ullah Saif MM (2020). Comparison of efficacy of dexamethasone and methylprednisolone in improving PaO_2_/FiO_2_ ratio among COVID-19 patients. Cureus.

[67] Rodriguez-Molinero A, Perez-Lopez C, Galvez-Barron C, Minarro A, Rodriguez Gullello EA, Collado Perez I, Mila Rafols N, Monaco EE, Hidalgo Garcia A, Ananos Carrasco G, Chamero Pastilla A, en representacion del grupo de investigadores para la C-dCSdlAPiG. Association between high-dose steroid therapy, respiratory function, and time to discharge in patients with COVID-19: cohort study. Med Clin. 2021;156(1):7–12. 10.1016/j.medcli.2020.08.003 10.1016/j.medcle.2020.08.001 PMC7691846 33263084

[68] Spagnuolo V, Guffanti M, Galli L, Poli A, Querini PR, Ripa M, Clementi M, Scarpellini P, Lazzarin A, Tresoldi M, Dagna L, Zangrillo A, Ciceri F, Castagna A, group CO-Bs, (2020). Viral clearance after early corticosteroid treatment in patients with moderate or severe COVID-19. Sci Rep.

[69] Lu X, Chen T, Wang Y, Wang J, Yan F (2020). Adjuvant corticosteroid therapy for critically ill patients with COVID-19. Crit Care.

[70] Hu Y, Wang T, Hu Z, Wang X, Zhang Z, Li L, Peng P (2020). Clinical efficacy of glucocorticoid on the treatment of patients with COVID-19 pneumonia: a single-center experience. Biomed Pharmacother.

[71] Zhu HM, Li Y, Li BY, Yang S, Peng D, Yang X, Sun XL, Zhang M (2020). Effect of methylprednisolone in severe and critical COVID-19: analysis of 102 cases. World J Clin Cases.

[72] Guo KJ, Zhao FC, Guo Y, Li FL, Zhu L (2014). The influence of age, gender and treatment with steroids on the incidence of osteonecrosis of the femoral head during the management of severe acute respiratory syndrome: a retrospective study. Bone Joint J.

[73] Nakamura J, Harada Y, Oinuma K, Iida S, Kishida S, Takahashi K (2010). Spontaneous repair of asymptomatic osteonecrosis associated with corticosteroid therapy in systemic lupus erythematosus: 10-year minimum follow-up with MRI. Lupus.

[74] Shibatani M, Fujioka M, Arai Y, Takahashi K, Ueshima K, Okamoto M, Yoshimura N, Hirota Y, Fukushima W, Kubo T (2008). Degree of corticosteroid treatment within the first 2 months of renal transplantation has a strong influence on the incidence of osteonecrosis of the femoral head. Acta Orthop.

[75] Griffith JF, Antonio GE, Kumta SM, Hui DS, Wong JK, Joynt GM, Wu AK, Cheung AY, Chiu KH, Chan KM, Leung PC, Ahuja AT (2005). Osteonecrosis of hip and knee in patients with severe acute respiratory syndrome treated with steroids. Radiology.

[76] Chan MH, Chan PK, Griffith JF, Chan IH, Lit LC, Wong CK, Antonio GE, Liu EY, Hui DS, Suen MW, Ahuja AT, Sung JJ, Lam CW (2006). Steroid-induced osteonecrosis in severe acute respiratory syndrome: a retrospective analysis of biochemical markers of bone metabolism and corticosteroid therapy. Pathology.

[77] Lopez Zuniga MA, Moreno-Moral A, Ocana-Granados A, Padilla-Moreno FA, Castillo-Fernandez AM, Guillamon-Fernandez D, Ramirez-Sanchez C, Sanchez-Palop M, Martinez-Colmenero J, Pimentel-Villar MA, Blazquez-Rosello S, Moreno-Sanchez JJ, Lopez-Vilchez M, Prior-Sanchez I, Jodar-Moreno R, Lopez Ruz MA (2021). High-dose corticosteroid pulse therapy increases the survival rate in COVID-19 patients at risk of hyper-inflammatory response. PLoS ONE.

[78] Callejas Rubio JL, Luna Del Castillo JD, de la Hera FJ, Guirao Arrabal E, Colmenero Ruiz M, Ortego Centeno N (2020). Effectiveness of corticoid pulses in patients with cytokine storm syndrome induced by SARS-CoV-2 infection. Med Clin.

[79] Liu F, Ji C, Luo J, Wu W, Zhang J, Zhong Z, Lankford S, Huang H, Lin F, Wang Y, Mo G, Hu X, Jiang T, Shao Y, Ji S, Zhang Y, Qin E, Mu J (2020). Clinical characteristics and corticosteroids application of different clinical types in patients with corona virus disease 2019. Sci Rep.

[80] Ruiz-Irastorza G, Pijoan JI, Bereciartua E, Dunder S, Dominguez J, Garcia-Escudero P, Rodrigo A, Gomez-Carballo C, Varona J, Guio L, Ibarrola M, Ugarte A, Martinez-Berriotxoa A, Cruces CSG (2020). Second week methyl-prednisolone pulses improve prognosis in patients with severe coronavirus disease 2019 pneumonia: an observational comparative study using routine care data. PLoS ONE.

[81] Sodhi N, Acuna A, Etcheson J, Mohamed N, Davila I, Ehiorobo JO, Jones LC, Delanois RE, Mont MA (2020). Management of osteonecrosis of the femoral head. Bone Joint J.

[82] Yildiz N, Ardic F, Deniz S (2008). Very early onset steroid-induced avascular necrosis of the hip and knee in a patient with idiopathic thrombocytopenic purpura. Intern Med.

[83] Fisher DE, Bickel WH (1971). Corticosteroid-induced avascular necrosis. A clinical study of seventy-seven patients. J Bone Joint Surg Am.

[84] Zhao R, Wang H, Wang X, Feng F (2017). Steroid therapy and the risk of osteonecrosis in SARS patients: a dose-response meta-analysis. Osteoporos Int.

[85] Pawar N, Vaish A, Vaishya R (2022). Core decompression and bone marrow aspirate concentrate injection for avascular necrosis (AVN) of the femoral head: a scoping review. J Clin Orthop Trauma.

[86] Birla V, Vaish A, Vaishya R (2021). Risk factors and pathogenesis of steroid-induced osteonecrosis of femoral head—a scoping review. J Clin Orthop Trauma.

[87] Vaishya R, Agarwal AK, Gupta NVV (2016). Sartorius muscle pedicle iliac bone graft for the treatment of avascular necrosis of femur head. J Hip Preserv Surg.

[88] Migliorini F, Maffulli N, Baroncini A, Eschweiler J, Tingart M (2022). Prognostic factors in the management of osteonecrosis of the femoral head: a systematic review. Surgeon.

[89] Yan Z, Hang D, Guo C, Chen Z (2009). Fate of mesenchymal stem cells transplanted to osteonecrosis of femoral head. J Orthop Res.

[90] Koo KH, Kim R, Ko GH, Song HR, Jeong ST, Cho SH (1995). Preventing collapse in early osteonecrosis of the femoral head. A randomised clinical trial of core decompression. J Bone Joint Surg Br.

[91] Stulberg BN, Davis AW, Bauer TW, Levine M, Easley K (1991). Osteonecrosis of the femoral head. A prospective randomized treatment protocol. Clin Orthop Relat Res.

[92] Hernigou P, Beaujean F, Lambotte JC (1999). Decrease in the mesenchymal stem-cell pool in the proximal femur in corticosteroid-induced osteonecrosis. J Bone Joint Surg Br.

[93] Gangji V, De Maertelaer V, Hauzeur JP (2011). Autologous bone marrow cell implantation in the treatment of non-traumatic osteonecrosis of the femoral head: five year follow-up of a prospective controlled study. Bone.

[94] Hauzeur JP, De Maertelaer V, Baudoux E, Malaise M, Beguin Y, Gangji V (2018). Inefficacy of autologous bone marrow concentrate in stage three osteonecrosis: a randomized controlled double-blind trial. Int Orthop.

[95] Sen RK, Tripathy SK, Aggarwal S, Marwaha N, Sharma RR, Khandelwal N (2012). Early results of core decompression and autologous bone marrow mononuclear cells instillation in femoral head osteonecrosis: a randomized control study. J Arthroplasty.

[96] Tabatabaee RM, Saberi S, Parvizi J, Mortazavi SM, Farzan M (2015). Combining concentrated autologous bone marrow stem cells injection with core decompression improves outcome for patients with early-stage osteonecrosis of the femoral head: a comparative study. J Arthroplasty.

[97] Zhao D, Cui D, Wang B, Tian F, Guo L, Yang L, Liu B, Yu X (2012). Treatment of early stage osteonecrosis of the femoral head with autologous implantation of bone marrow-derived and cultured mesenchymal stem cells. Bone.

[98] Pepke W, Kasten P, Beckmann NA, Janicki P, Egermann M (2016). Core decompression and autologous bone marrow concentrate for treatment of femoral head osteonecrosis: a randomized prospective study. Orthop Rev.

[99] Wu ZY, Sun Q, Liu M, Grottkau BE, He ZX, Zou Q, Ye C (2020). Correlation between the efficacy of stem cell therapy for osteonecrosis of the femoral head and cell viability. BMC Musculoskelet Disord.

[100] Kang JS, Suh YJ, Moon KH, Park JS, Roh TH, Park MH, Ryu DJ (2018). Clinical efficiency of bone marrow mesenchymal stem cell implantation for osteonecrosis of the femoral head: a matched pair control study with simple core decompression. Stem Cell Res Ther.

[101] Li X, Xu X, Wu W (2014). Comparison of bone marrow mesenchymal stem cells and core decompression in treatment of osteonecrosis of the femoral head: a meta-analysis. Int J Clin Exp Pathol.

[102] Papakostidis C, Tosounidis TH, Jones E, Giannoudis PV (2016). The role of "cell therapy" in osteonecrosis of the femoral head. A systematic review of the literature and meta-analysis of 7 studies. Acta Orthop.

[103] Yuan HF, Zhang J, Guo CA, Yan ZQ (2016). Clinical outcomes of osteonecrosis of the femoral head after autologous bone marrow stem cell implantation: a meta-analysis of seven case-control studies. Clinics.

[104] Xu S, Zhang L, Jin H, Shan L, Zhou L, Xiao L, Tong P (2017). Autologous stem cells combined core decompression for treatment of avascular necrosis of the femoral head: a systematic meta-analysis. Biomed Res Int.

[105] Wang Z, Sun QM, Zhang FQ, Zhang QL, Wang LG, Wang WJ (2019). Core decompression combined with autologous bone marrow stem cells versus core decompression alone for patients with osteonecrosis of the femoral head: a meta-analysis. Int J Surg.

[106] Hernigou P, Dubory A, Homma Y, Guissou I, Flouzat Lachaniette CH, Chevallier N, Rouard H (2018). Cell therapy versus simultaneous contralateral decompression in symptomatic corticosteroid osteonecrosis: a thirty year follow-up prospective randomized study of one hundred and twenty five adult patients. Int Orthop.

[107] Migliorini F, Maffulli N, Eschweiler J, Tingart M, Baroncini A (2020). Core decompression isolated or combined with bone marrow-derived cell therapies for femoral head osteonecrosis. Expert Opin Biol Ther.

[108] Eisenschenk A, Lautenbach M, Schwetlick G, Weber U (2001). Treatment of femoral head necrosis with vascularized iliac crest transplants. Clin Orthop Relat Res.

[109] Judet H, Gilbert A (2001). Long-term results of free vascularized fibular grafting for femoral head necrosis. Clin Orthop Relat Res.

[110] Mukisi-Mukaza M, Manicom O, Alexis C, Bashoun K, Donkerwolcke M, Burny F (2009). Treatment of sickle cell disease's hip necrosis by core decompression: a prospective case-control study. Orthop Traumatol Surg Res.

[111] Rajagopal M, Balch Samora J, Ellis TJ (2012). Efficacy of core decompression as treatment for osteonecrosis of the hip: a systematic review. Hip Int.

[112] Mont MA, Fairbank AC, Krackow KA, Hungerford DS (1996). Corrective osteotomy for osteonecrosis of the femoral head. J Bone Joint Surg Am.

[113] Sakano S, Hasegawa Y, Torii Y, Kawasaki M, Ishiguro N (2004). Curved intertrochanteric varus osteotomy for osteonecrosis of the femoral head. J Bone Joint Surg Br.

[114] Hernigou P, Flouzat-Lachaniette CH, Delambre J, Poignard A, Allain J, Chevallier N, Rouard H (2015). Osteonecrosis repair with bone marrow cell therapies: state of the clinical art. Bone.

[115] Lau RL, Perruccio AV, Evans HM, Mahomed SR, Mahomed NN, Gandhi R (2014). Stem cell therapy for the treatment of early stage avascular necrosis of the femoral head: a systematic review. BMC Musculoskelet Disord.

[116] Migliorini F, Maffulli N, Baroncini A, Eschweiler J, Tingart M, Betsch M (2021). Failure and progression to total hip arthroplasty among the treatments for femoral head osteonecrosis: a Bayesian network meta-analysis. Br Med Bull.

[117] Talathi NS, Kamath AF (2018). Autologous stem cell implantation with core decompression for avascular necrosis of the femoral head. J Clin Orthop Trauma.

[118] Malizos KN, Karantanas AH, Varitimidis SE, Dailiana ZH, Bargiotas K, Maris T (2007). Osteonecrosis of the femoral head: etiology, imaging and treatment. Eur J Radiol.

[119] Lavernia CJ, Sierra RJ, Grieco FR (1999). Osteonecrosis of the femoral head. J Am Acad Orthop Surg.

[120] Gangji V, Hauzeur JP (2010). Treating osteonecrosis with autologous bone marrow cells. Skeletal Radiol.

[121] Jones KB, Seshadri T, Krantz R, Keating A, Ferguson PC (2008). Cell-based therapies for osteonecrosis of the femoral head. Biol Blood Marrow Transplant.

[122] Maestro-Paramio L, Garcia-Rey E, Bensiamar F, Saldana L (2021). Osteoblast function in patients with idiopathic osteonecrosis of the femoral head: implications for a possible novel therapy. Bone Joint Res.

[123] Zhu S, Zhang X, Chen X, Wang Y, Li S, Qian W (2021). Comparison of cell therapy and other novel adjunctive therapies combined with core decompression for the treatment of osteonecrosis of the femoral head : a systematic review and meta-analysis of 20 studies. Bone Joint Res.

